# The concept of effective coverage radius use of the unlicensed high-frequency range in the operation of the 5G network

**DOI:** 10.1016/j.heliyon.2024.e39825

**Published:** 2024-10-25

**Authors:** Viacheslav Kovtun, Krzysztof Grochla, Elena Zaitseva, Vitaly Levashenko

**Affiliations:** a Internet of Things Group, Institute of Theoretical and Applied Informatics Polish Academy of Sciences, Gliwice, Poland; b Department of Informatics, University of Žilina, Zilina, Žilina, Slovakia

**Keywords:** 5G network, Unlicensed frequency range, Quality of service, Queuing markov mode, Coating density, Probability of session loss

## Abstract

Economic expediency encourages mobile operators to deploy 5G networks in places with a high concentration of speed-demanding subscribers. In such conditions, sharp fluctuations in the volume of traffic with regulated requirements for the quality of service are inevitable. Note that 5G operates in the millimeter range. Accordingly, the quality of traffic service is affected both by the number of subscribers simultaneously initiating requests from one sector of coverage, and by the appearance of obstacles opaque to radio radiation in the space between the subscriber device and the base station. Effective smoothing of 5G traffic fluctuations, taking into account these disturbing factors, is an urgent task. The goal of this research is to evaluate the service quality parameters in a target area characterized by a specific user density. It takes into account that if the declared QoS requirements for connection speed for users in a network segment deployed within the licensed frequency range for 5G are not met, they can utilize a network segment deployed in the unlicensed high-frequency range for 5G under conditions of free competition. The metric being studied is the probability of session loss in the licensed network segment and the achievable transmission speed in the unlicensed network segment. Based on this, a method for assessing the density of base station deployment in the unlicensed network segment necessary to support the specified user density in the licensed network segment with defined QoS guarantees in terms of bandwidth is formalized. The experiment results showed that the probability of losing sessions with regulated requirements for the quality of service in both network segments, in addition to the base station placement density and subscriber devices, is significantly affected by the minimum data transfer rate, the intensity of obstacles, and the value of the Contention Window.

## Nomenclature

$NS_{L}$: is the first of two network segments on a single hardware platform that deployed in a licensed frequency range
$NS_{\bar{L}}$: is the second of two network segments on a single hardware platform that deployed in an unlicensed frequency range
$W_{L}$: is the channel width for a $NS_{L}$ network segment
$W_{\bar{L}}$: is the channel width for a $NS_{\bar{L}}$ network segment
$h_{BS}$: is a height on which 5G base stations are mounted
$\eta_{BS}$: is a density at which 5G base stations should cover a target area $R^{2}$
$S_{L\bar{L}}$: is the type of subscriber device capable of being served in both network segments
$S_{\bar{L}}$: is the type of subscriber device capable of being served only in $NS_{\bar{L}}$ network segments
$\mu_{L\bar{L}}$: is a density of location of subscriber devices of the $S_{L\bar{L}}$ type in the target area $R^{2}$
$\mu_{\bar{L}}$: is a density of location of subscriber devices of the $S_{\bar{L}}$ type in the target area $R^{2}$
$h_{S}$: is a height on which all subscriber devices are located
$S_{CW}$: is a the size of the Contention Window
N: is the maximum number of reconnections
R(x): is the Signal-to-Interference-plus-Noise Ratio for a subscriber device located at a distance χ from the base station
$P_{S}$: is the transmission power of the subscriber device;
$G_{BS}$, $G_{S}$: are the gain coefficients of the antenna arrays of the base station and subscriber device, respectively
$L_{I}$: is the interference limit
L(x): is propagation loss
$S_{0}$: is the noise power spectral density
${Β}_{W}$: is the working bandwidth
L(x): is the propagation loss
$F_{W}$: is the operating frequency
$a_{i}$, $\xi_{i}$: are the propagation coefficients
$p_{O}\left(x\right)$: is the probability that the space between the subscriber device and the base station will be blocked at a distance of χ
K: is a generalizing factor
$h_{P}$, $r_{P}$: are the height and radius of the cylinder that is a pedestrian interpretation
$\eta_{P}$: is the pedestrian density
ϕ: is the Half-Power-Beam-Width of the subscriber antenna array
$\varphi_{3\text{dB}}$: is the angle at which the value of the radiated power is 3 dB less than the maximum
$\varphi_{\max}$: is the position of the maximum of the antenna array
α: is the azimuthal angle of the physical orientation of the antenna array
$G_{A}$: the average gain for the antenna array
$n_{A}$: is the number of antenna array elements
$V_{\min}$: is the minimum acceptable speed declared in the Quality of Service for $S_{L\bar{L}}$
$r_{NS_{L}}$, $r_{NS_{\bar{L}}}$: are the effective coverage radii for network segments $NS_{L}$, $NS_{\bar{L}}$, respectively
$r_{BS,BS}$: is the distance between base stations in the investigated 5G network
$r_{BS,S}$: is the coverage of the base station in the 5G network
$R_{low}$: is the Signal-to-Noise Ratio (SNR) at a distance $r_{BS,BS}$
$K_{2}$: is the SNR corresponds to the lowest possible Modulation-and-Coding Scheme
$L_{R}$: is the signal fading limit, erfc is the additional Gaussian error function
$\sigma_{L_{R}}$: is a signal fading limit standard deviation
$p_{BS}$: is a cell boundary coverage probability
$w_{r}\left(y\right)$: is the distribution density of the distance y between the subscriber device and the base station on the plane
$y=\gamma_{i}\left(x\right)=1/\psi \left(y\right)$: are inverse functions presented in the expression (6), $h_{BS}-h_{S}<y<\sqrt{r_{NS_{L}}^{2}+h_{BS}^{2}-2h_{BS}h_{S}+h_{S}^{2}}$
$W_{d}\left(y\right)$: is the SNR without fading
d: is the distance between $S_{L\bar{L}}$ and the base station in three-dimensional space
$\psi_{SNR,dB}\left(d\right)$: is the inverse function of $W_{d}\left(y\right)$
а: is a coefficient that summarizes all gains and losses (except propagation losses and fluctuations caused by fading)
ξ: is a coefficient that characterizes propagation losses
$W_{SNR,dB}\left(y\right)$: is the SNR distribution function
$W_{SNR,Att,dB}$: is the SNR distribution function with attenuation taken into account
$\sigma_{Att}$: is a fading standard deviation
u: **g** is the increase in the value of y
Φ: is the Laplace function
$b=r_{NS_{L}}^{2}+{\left(h_{BS}-h_{S}\right)}^{2}$: is a parameter
$\sigma_{Att,O}$, $\sigma_{Att,\bar{O}}$: are options for determining the dispersion $\sigma_{Att}$ with and without taking into account the presence of obstacle
$E\left(R_{l}\right)$: is the average spectral efficiency of the session in the investigated 5G network
R(y): is the SINR for the subscriber device $S_{L\bar{L}}$ located at a distance l from the base station
η: is an intensity of session activation requests in the investigated 5G network
$\pi r_{NS_{L}}^{2}$: is the coverage circle for the network segment $NS_{L}$ (see Section 2.3)
$p_{T}$: is the probability that the session $S_{L\bar{L}}$ is active;
$\eta_{S_{L\bar{L}}}$: is the intensity with which $S_{L\bar{L}}$ generates requests for session activation
$\eta_{1}$: is the intensity of generating requests for activating sessions of the first type
$\eta_{2}$: is the intensity of generating requests for activating sessions of the second type
$\eta_{L}$: is the intensity of incoming requests to the network segment $NS_{L}$
$\eta_{\bar{L}}$: is the intensity of incoming requests to the network segment $NS_{\bar{L}}$
$q_{2}$: is the probability of forwarding a session of the second type to the network segment $NS_{\bar{L}}$
$q_{1}$: us the probability of losing a session of the first type
$A_{R}$: is the amount of communication resources
$N_{S}$: is a process of sessions servicing
$N_{S}$: represents the number of serviced subscriber devices $S_{L\bar{L}}$
μ: is a parameter of exponentially distributed service duration
$a_{R}$: is the amount of communication resources needed to service the session
$p_{s,a}$: is the probability that the amount of communication resources equal to а is required to support a session of type s={1,2}
$p_{a,L}=\left(\beta_{1}p_{1,a}+\beta_{2}p_{2,a}\right)/\beta$: is the resource requirements in an aggregated flow of incoming requests, $\beta_{s}=\eta_{s}/\mu$, $\beta =\sum_{s}\beta_{s}$, s={1,2}
$X\left(t\right)=\left(N_{S}\left(t\right),A_{R}\left(t\right)\right)$: is the stochastic process that describes the behavior of the system
$N_{S}\left(t\right)$: is the number of sessions supported by the system
$A_{R}\left(t\right)=\left\{a_{i}\left(t\right)\right\}$, $i=\bar{1,N_{S}}$: is the number of communication resources reserved for the service of the і th active session at a time t
$P_{s}\left(a_{R}\right)$: is the stationary probability that the system serves s sessions, for which the amount of communication resources equal to $a_{R}$ is spent
$p_{a_{R},L}^{\left(s\right)}$: is a recursively computed convolution operation of the ${\left\{p_{a_{R},L}\right\}}_{a_{R}>0}$ distribution
$H\left(N_{S},a_{R}\right)$: is the recursively computed function
$p_{i,\bar{L}}$: id the distribution of requirements for the amount of resources necessary to support sessions redirected to the network segment $NS_{\bar{L}}$
$p_{ST}$: is the probability of successful transmission for devices $S_{L\bar{L}}$, $S_{\bar{L}}$
$p_{D}$: is the probability of distortions caused by the simultaneous operation of devices $S_{L\bar{L}}$, $S_{\bar{L}}$
$p_{O}$: is the probability of an obstacle appearing in the space between the subscriber device and the base station
$F=\left\{F_{n},n\geq 0\right\}$: is a Markov chain that presents the corresponding procedure for random access to the transmission medium
$F_{n}$: is the parameter that represents the number of unsuccessful data transmission attempts by the subscriber device since the last successful attempt, $F_{n}\in \left[0,\tau \right]$
$\rho_{і}$: are the stationary probabilities of realization of the corresponding states of this Markov process F
$q_{L\bar{L}}$, $q_{\bar{L}}$: are the probabilities that subscriber devices $S_{L\bar{L}}$, $S_{\bar{L}}$, respectively, make requests to activate sessions at the same time;
$z_{j}$: is the average number of tacts of $\left\{F_{n}\right\}$ being in state j, $j=\bar{0,\tau }$
$n_{L\bar{L}}$, $n_{\bar{L}}$: are the number of competing sessions for the $S_{L\bar{L}}$ $S_{\bar{L}}$ subscriber devices, respectively
$P_{L\bar{L}}$, $P_{\bar{L}}$: are the resulting probability of data transmission for $S_{L\bar{L}}$, $S_{\bar{L}}$, respectively, in the investigated 5G network
$V_{\bar{L},j}^{L\bar{L}}$: is the speed of a session with spectral efficiency $e_{j}$
$E\left(V_{\bar{L}}^{L\bar{L}}\right)$: is the average speed for the $S_{L\bar{L}}$-sessions supported in the network segment $NS_{\bar{L}}$
Q: is the resulting probability of request consumption in the investigated 5G network

## 1 Introduction

### 1.1 Relevance of the research

The transition to 5G systems in the cellular market heralds a promising era of significantly improved data transfer speed and reduced latencies at a wireless access interface, coupled with the flexibility of software-enabled dynamic reconfiguration on request. As a result, 5G rollouts are poised to meet the growing demands of various applications. By 2025, it is anticipated that there will be at least 2.8 billion 5G subscriptions, with smart devices potentially constituting up to 80 % of these connections [[1], [2], [3]]. These evolving needs are mainly driven by the growing prevalence of video-based offerings, expected to make up approximately 75 % of the total network traffic by 2025. Furthermore, the rise of innovative applications includes, different “extreme” types of virtual reality [4], Internet of drones [5], and so on.

As a reaction to the aforementioned developments, mobile carriers are gearing up to initially embrace New Radio (NR) technology solutions within a sub-6 GHz frequency range [6]. With the specifications for NR, both independent and integrated options, being solidified in 5G R.15 [1,7], standardization agencies (3GPP, ITU) are presently focusing on the employment of, 28 GHz, 38 GHz, and 73 GHz higher frequency ranges. Nevertheless, a widespread propagation of throughput-intensive applications and services in the future could potentially result in spectrum scarcity and traffic overload, particularly in widely populated public places, even for NR-supported systems.

Numerous data-intensive applications are anticipated to be utilized in widely populated areas. In the mentioned environments, only millimeter-wave communication technology holds the potential to effectively handle substantial traffic loads [2]. However, contemporary mobile traffic demands exhibit significant temporal fluctuations [8]. Furthermore, known studies have indicated that these temporal fluctuations have started to be increasingly unpredictable and densely concentrated in contrast to prior years [9]. This shift can be attributed, in part, to the proliferation of advanced applications like virtual reality gaming. This situation is unlikely to revise even with the NR usage, as these mobile access systems are anticipated to remain isolated and high-throughput enclaves [10,11].

### 1.2 State-of-the-Art

Presently, standardization agencies and researchers are actively seeking a methodological solution to account for challenges posed by temporal fluctuations in mobile data transfer flow. In this context, we investigate a promising approach to alleviate the effect of traffic variability, namely, redirecting it to the unlicensed millimeter frequency range. By aggregating point-to-point connections through licensed and unlicensed millimeter frequency ranges, mobile subscriber devices gain the potential to redirect redundant traffic while simultaneously reducing levels of traffic variability.

As we have seen, the scientific community is actively exploring the effective use of high-frequency spectrum for the implementation of digital communications. This is confirmed, for example, by review articles such as [[12], [13], [14], [15]]. Now let's move on to analyzing works that are directly related to the topic of our research.

In [16,17], researchers explored the notion of integrating licensed and unlicensed millimeter frequency ranges. Their investigation focused on the concurrent existence of these two ecosystems, particularly About downlink data speeds. They compared three different scenarios: the exclusive use of IEEE 802.11, the simultaneous operation of 802.11 and NRU, and the NRU operating alone. The findings revealed that the utilization of unlicensed ranges by NRU subscriber devices could significantly impair the data rate performance of 802.11 subscriber devices.

Given the potential for increased interference associated with NRU operation, recent research has shifted its focus toward establishing effective coexistence mechanisms between NRU and 802.11 systems. This research is encompassed in Refs. [18,19], which explore aspects like duty cycles and random access. Reference [18] lays out the overarching design objectives for concurrent existence technologies in these systems operating within the 60 GHz range. It delves into architectural details regarding 5G NRU to facilitate spectrum sharing in unlicensed frequency ranges. In contrast, reference [19] introduces the Listen-Before-Receive (LBR) mechanism for joint spectrum utilization and evaluates its ability to contribute to equitable coexistence among various mobile technologies in the unlicensed frequency ranges. Furthermore, reference [20] presents the Reinforcement learning-used Listen-Before-Talk (ReLBT) technique to enhance fairness. It does so by incorporating a more useful channel surveillance scenario that dynamically adjusts the Contention Window (CW) to optimize communication resource usage and coexistence productivity.

In a more recent study, detailed in Ref. [21], authors introduced a hybrid channel access technique that blends both LBT and non-LBT approaches. This innovative method switches to LBT when the channel experiences extreme congestion, while it employs non-LBT access when a medium is comparatively unoccupied. Comprehensive simulations confirmed that this proposed technique can deliver higher subscriber-perceived capacity and reduced latency in comparison to scenarios where LBT is consistently enabled. Reference [8,22] further explored performance comparisons between combinations of directional and omnidirectional channel access strategies alongside both LBT and LBR technologies. These evaluations took into account realistic factors such as millimeter frequency range antenna array patterns, indoor path loss models proposed by the 3GPP, and fixed back-off procedures. The findings showcased that a directional approach with a combination of directional LBT and directional LBR, in addition to omnidirectional LBT, outperformed other configurations regarding measures like total data speed, average data speed, and minimum data speed. Furthermore, directional LBT was identified as the preferred choice for achieving fairness. In Ref. [23], the authors introduced a framework that displays the impact of deficient spectrum perception and the implementation of multiple cell and multiple level LBT technologies onto key productivity indicators.

Some productivity evaluations focusing on other coexistence options between Wi-Fi and 4G LTE, within the context of Licensed Assisted Access (LAA) technologies, have been reported to date. Notably, prior studies have employed LBT and duty-cycle strategies for enabling the coexistence of Wi-Fi and 4G LTE (LAA), as documented in Refs. [24,25]. In Ref. [24], the authors examined the performance of Wi-Fi when sharing a channel with LAA transmissions governed by duty cycles. This study involved the consideration of one 4G LTE base station and one Wi-Fi access point, with LAA devices restricted to transmitting their signals only during pre-determined duty cycles. In contrast, Wi-Fi base stations were granted to compete for access to a shared channel devoid of any coordination with the LAA system, and they possessed no previous knowledge regarding operating-cycle parameters. Additionally, reference [25] introduced coexistence scenarios designed to enhance the interactions between Wi-Fi and 4G LTE systems, with the goal of potential improvements in forthcoming New Radio (NR) networks.

Reference [26] utilized effective capacity concepts to evaluate the statistical Quality of Service (QoS) assurances for LTE sessions. To catch Data transmission conflicts, casual back-offs, and the impact of lossy channels in heterogeneous wireless networks, the study created a four-state semi-Markov model. Additionally, it provided a closed-form expression for assessing the investigated system's effective capacity. Conversely, the model presented in Ref. [27] focused on the coexistence of Wi-Fi and 4G LTE systems, particularly within the context of channel access controlled by LBT.

Reference [28] extended research, which had previously been outlined in Ref. [26], to explore the interaction between NRU and Wi-Fi systems. The authors introduced the novel LBT protocol, called cooperative LBT, which includes zero-forcing precoding for multiple user interference. Reference [29] delved into characterizing prominent interference origins and provided analytical formulas to describe the dimensionally averaged productivity of the typical communication link. Meanwhile, the study documented in Ref. [30] investigated the duty-cycle mechanisms introduced by LAA and demonstrated their potential to offer QoS definitions and provide equitable resource allocation across Wi-Fi and LTE systems. This model was subsequently expanded to accommodate the elastic type of traffic in Ref. [13].

It is also worth mentioning the implementation of Intelligent Networking into the technological foundation of 5G networks [[31], [32], [33]]. Undoubtedly, this is an important stage in the development of wireless technologies. However, the application of Intelligent Networking is accompanied by both significant advantages and certain challenges. The integration of artificial intelligence and machine learning into network management allows for the automation of many processes, including traffic optimization, failure prediction, and overall network efficiency improvement. This enhances the quality of service for users and reduces operational costs for operators. However, critics point out risks related to cybersecurity and data privacy, which are exacerbated by the autonomy of such systems. Additionally, the complexity of implementing and managing highly intelligent networks requires significant investments and deep infrastructure changes, which could be a challenge for less prepared operators.

The general drawback of the mentioned studies is their "laboratory" orientation. The 5G network operates in the millimeter range. Accordingly, the quality of traffic service is affected both by the number of subscribers simultaneously initiating requests from one sector of coverage, and by the appearance of obstacles opaque to radio radiation in the space between the subscriber device and the base station. The influence of these disturbing factors should be taken into account in the models that describe the functioning of a real 5G network. We also note that the vast majority of the mentioned studies consider the one located around the frequency of 60 GHz as an unlicensed high-frequency range. In practice, this means that engineers will have to coordinate the operation of 5G equipment with other communication technologies (Wi-Fi, WiGig, etc.). Of course, compatibility with the IEEE 802.11 standard is declared for these technologies, but the integration of different interfaces is never easy or problem-free Instead, if we focus on the currently unlicensed frequency range of around 38 GHz, even antenna arrays for base stations can be chosen unified (see, for example, references [34,35]).

### 1.3 Main attributes of the research

In this section we will present the main elements that determine the orientation, structure, and novelty of the presented research.

The ***object*** of the research is the session control process in the network segments of the 5G network deployed in the licensed and unlicensed frequency ranges of the millimeter band.

The ***subject*** of research includes the terms and methods of mathematical modeling, queuing theory, probability theory, and functional analysis.

The ***aim*** of the research is formulated as follows: to formalize the functional dependence between the probabilities of losing sessions with regulated conditions regarding the quality of service in network segments of the 5G network deployed in licensed and unlicensed frequency bands, and the base stations placement density to support the traffic of a corresponding density of dual frequency range subscriber devices for the target coverage area.

Now let's define the ***tasks*** of the research.

- to formalize the parametric space for an adequate description of the research object,
- to formalize the concepts that characterize the peculiarities of signal propagation between the subscriber device and the base station in the investigated dual frequency ranges 5G network,
- formalize the approach to assessing the resource consumption of a session with a target subscriber device in the investigated dual frequency ranges 5G network,
- to formalize the concept of 5G network operation in licensed and unlicensed frequency bands,
- implement a numerical experiment, the results of which will justify the effectiveness of the created mathematical apparatus.

Now let's formulate the ***main contribution*** of the research. The goal of this research is to evaluate the service quality parameters in a target area characterized by a specific user density. It takes into account that if the declared QoS requirements for connection speed for users in a network segment deployed within the licensed frequency range for 5G are not met, they can utilize a network segment deployed in the unlicensed high-frequency range for 5G under conditions of free competition. The metric being studied is the probability of session loss in the licensed network segment and the achievable transmission speed in the unlicensed network segment. Based on this, a method for assessing the density of base station deployment in the unlicensed network segment necessary to support the specified user density in the licensed network segment with defined QoS guarantees in terms of bandwidth is formalized. The numerical study conducted showed that, in addition to user density in the licensed and unlicensed network segments, the investigated characteristics are influenced by the size of the contention window, the density of obstacles (other users) in the signal propagation path, and the minimum connection speed specified in the QoS. The obtained experimental results allow the conclusion that the proposed approach can significantly increase the achievable speed of user sessions; however, this requires a sufficiently dense deployment of the unlicensed network segment.

The hightlights of the recearch are:

- the principles that define the unique characteristics of signal propagation between subscriber devices and base stations in the dual-frequency bands of the 5G network under investigation.

- the method for evaluating the resource consumption of a session involving a target subscriber device within the dual-frequency bands of the 5G network under study.
- the operational concept of a 5G network utilizing both licensed and unlicensed frequency bands.

## 2 Models and methods

### 2.1 Statement of the research

Suppose that the 5G base station supports, in particular, NRU, Network Slicing (NS), and Carrier Aggregation (CA) technologies, which allows for the simultaneous operation of two network segments on a single hardware platform: $NS_{L}$, $NS_{\bar{L}}$. The first of them is deployed in a licensed frequency range with an operating frequency of 28 GHz and a channel width of $W_{L}=200$ MHz. The second network segment is deployed in an unlicensed frequency range with an operating frequency of 38 GHz and a channel width of $W_{\bar{L}}=160$ MHz.

Suppose that a set of such 5G base stations is mounted at a height $h_{BS}$ and designed to cover a square meter of a target area $R^{2}$ with a density $\eta_{BS}$ according to a point Poisson process. Within the coverage of the deployed 5G network, the functioning of two types of subscriber devices is assumed. The first type of subscriber device $S_{L\bar{L}}$ is capable of being served in both network segments (targeting the 28 GHz and 38 GHz frequency ranges, respectively). The second type of subscriber device $S_{\bar{L}}$ can be served only in the second network segment (focusing on the 38 GHz frequency range). The location of subscriber devices is also described by a point Poisson process with densities $\mu_{L\bar{L}}$ and $\mu_{\bar{L}}$, respectively. At the same time, we consider that all subscriber devices are located at the height $h_{S}$, $h_{S}\ll h_{BS}$.

The method of interaction of the subscriber device $S_{L\bar{L}}$ with the segmented communication space of the investigated 5G network is determined on the side of the first. If when trying to connect to a network segment $NS_{L}$, $S_{L\bar{L}}$ it does not receive guaranteed quality of service, then it connects to the network segment $NS_{\bar{L}}$. If it also did not receive the guaranteed quality of service in the network segment $NS_{\bar{L}}$, then it stops trying to connect to the wireless network for a certain time.

Note that when trying to connect to $NS_{\bar{L}}$, both $S_{L\bar{L}}$ $S_{\bar{L}}$ devices use the LBT technique. At the same time, the NRU technology uses the Channel Observation-Based mechanism with a Contention Window (CW) and the implementation principle of the delay counter recommended by 3GPP for LAA technology (the initial size of the Contention Window $S_{CW}$ is 632, and the maximum number of reconnections is N). The subscriber device, when trying to connect to the investigated 5G network, generates a random integer number of delay clocks on the segment $\left[1,S_{CW}\right]$ according to a uniform distribution. The value of the delay counter is decremented by one every clock cycle where the channel is considered free. If the channel is considered busy, the counter is paused and channel listening continues. When the value of the delay counter reaches one, the subscriber device starts the process of transmitting the data packet. There are the following options to complete this process:

- the data packet was successfully transmitted,
- transmission failure for a technological reason (conflict with the same type of process activated by another subscriber device),
- transmission failure for a physical reason (an obstacle opaque to radio radiation appeared in the space between the subscriber device and the base station).

If the transfer is successful, the delay counter is reset to its initial value. Otherwise, the maximum value of the counter is doubled. The mentioned failures can occur only when an obstacle or several subscriber devices that simultaneously initiate transmission processes appear in the coverage sector of the directional antenna array of the base station.

### 2.2 Concepts that characterize the characteristics of signal propagation between the subscriber device and the base station in the investigated 5G network

In the previous section, we established that the result of the communication process in the investigated 5G network is affected by.

- features of radio signal propagation on $R^{2}$,
- features of antenna array orientation,
- the specificity of taking into account the possibility of overlapping the space between the subscriber device and the base station. Let us present these points in the form of relevant analytical concepts.

All technologies mentioned in Section 2.1 operate in the millimeter range. Accordingly, the Signal-to-Interference-plus-Noise Ratio (SINR) for a subscriber device located at a distance from the base station is described by the expression

(1) $$R\left(x\right)=\frac{P_{S}G_{BS}G_{S}}{L\left(x\right)\left(S_{0}{Β}_{W}+L_{I}\right)}\text{,}$$

where χ is the distance between the subscriber device and the base station; $P_{S}$ is the transmission power of the subscriber device; $G_{BS}$, $G_{S}$ are the gain coefficients of the antenna arrays of the base station and subscriber device, respectively; $L_{I}$ is the interference limit; L(x) is propagation loss; $S_{0}$ is the noise power spectral density; ${Β}_{W}$ is the working bandwidth.

For a given density of deployment of base stations of the investigated 5G network, we can estimate the value of interference using recognized models based on the mathematical apparatus of stochastic geometry. According to the 3GPP Urban Micro (UMi) street canyon model [36], the propagation loss L(x) measured in dB is determined from the ratio

(2) $$L\left(x\right)=\left\{ \begin{array}{l} 21\;\log\left(x\right)+20\;\log\;F_{W}+32.4\forall \;\text{case}1\left(there\;is\;no\;obstacle\right),\\ 40\;\log\left(x\right)+20\;\log\;F_{W}+32.4\forall \;\text{case}2\left(there\;is\;an\;obstacle\right), \end{array}\right.$$

where $F_{W}$ is the operating frequency in GHz; case1: if there is no obstacle in the space between the subscriber device and the base station; case2: if there is an obstacle in the space between the subscriber device and the base station.

For our case, the propagation loss function (2) can be approximated on a linear scale by a model of the form $a_{i}x^{-\xi_{i}}$, where $a_{i}$, $\xi_{i}$ are the propagation coefficients. Let us introduce sets $\left(a_{1},\xi_{1}\right)$, $\left(a_{2},\xi_{2}\right)$ that correspond to case1, case2. As a result, we get: $a=a_{1}=a_{2}=10^{2\;\mathrm{lg}\;F_{W}+3.24}$, $\xi_{1}=2.1$, $\xi_{2}=3.19$. Therefore, the expression (1) can be redefined:

(3) $$R\left(\chi \right)=K\left(x^{-\xi_{1}}\left(1-p_{o}\left(x\right)\right)+x^{-\xi_{2}}p_{O}\left(x\right)\right)\text{,}$$

where $p_{O}\left(x\right)$ is the probability that the space between the subscriber device and the base station will be blocked at a distance of χ; $K=\left(P_{S}G_{BS}G_{S}\right)/\left(a\left(S_{0}B_{W}+L_{I}\right)\right)$ is a generalizing factor.

Let us pay attention to the phenomenon of obstacles in the path of radio signal propagation in the space between the subscriber device and the base station. Note that stationary obstacles are static and their impact on the process of information transfer in the investigated 5G network can be taken into account by introducing the appropriate correction coefficients. The stochastic influence of dynamic obstacles (pedestrians, vehicles) is more difficult to take into account. In the first approximation, we will use the pedestrian mobility model [37]. This model assumes that pedestrians move along $R^{2}$ in arbitrary directions with a speed v mps and an exponentially distributed path length with mean ρ m. From the point of view of an obstacle to radio signals, the pedestrian is interpreted as a cylinder of height $h_{P}$ and radius $r_{P}$. With such input data, the probability that the space r between the subscriber device and the base station will be blocked by a pedestrian is characterized by an expression derived from Ref. [37]:

(4) $$p_{O}\left(r\right)=1-\exp\left(-2\eta_{P}r_{P}\left(r_{S,BS}\left(h_{P}-h_{S}\right)/\left(h_{BS}-h_{S}\right)+r_{P}\right)\right)\text{,}$$

where $\eta_{P}$ is the pedestrian density.

Finally, let's pay attention to the model of the focused antenna array. In the following, we will assume that the directional pattern of the antenna array represents a conical zone with an angle ϕ that coincides with the Half-Power-Beam-Width (HPBW) of the antenna array [38]. As is known, the HPBW of the antenna array is proportional to the number of elements: $\phi =2\left|\varphi_{\max}-\varphi_{3\text{dB}}\right|$, where $\varphi_{3\text{dB}}$ is the angle at which the value of the radiated power is 3 dB less than the maximum; $\varphi_{\max}=\arccos\left(-\alpha /\pi \right)$ is the position of the maximum of the antenna array, where α is the azimuthal angle of the physical orientation of the antenna array ($\varphi_{\max}=\pi /2$ for α=0). Based on HPBW, the average gain for the antenna array is expressed as

(5) $$G_{A}=\frac{1}{\varphi_{3\text{dB}}^{+}-\varphi_{3\text{dB}}^{-}}\int_{\varphi_{3\text{dB}}^{-}}^{\varphi_{3\text{dB}}^{+}}\frac{\sin\left(n_{A}\pi \;\cos\left(\varphi \right)/2\right)}{\sin\left(\pi \;\cos\left(\varphi \right)/2\right)}d\varphi \text{,}$$

where $n_{A}$ is the number of antenna array elements, $\varphi_{3\text{dB}}^{\pm }=\arccos\left(-\alpha \pm 2.78/\left(\pi n_{A}\right)\right)$.

### 2.3 Estimation of the resource consumption of a session with a target subscriber device in the investigated 5G network

Assume that $S_{L\bar{L}}$ generates elastic traffic demands, that is, subscriber devices always have data to transmit. At the same time, the minimum acceptable speed $V_{\min}$ is declared in the Quality of Service (QoS) for $S_{L\bar{L}}$. Naturally, as the $S_{L\bar{L}}$ moves away from the base station, the amount of communication resources that the 5G network spends on providing $V_{\min}$ increases.

For subscriber devices $S_{\bar{L}}$, which are served only in the network segment $NS_{\bar{L}}$, the criterion for choosing a base station is the reception power of the reference signal. In this context, by default $S_{\bar{L}}$ communicates with the nearest base station, because the QoS conditions $S_{\bar{L}}$ are not formalized. Instead, by default $S_{L\bar{L}}$ contacts the nearest base station for service purposes in the network segment $NS_{L}$. However, if the received communication characteristics do not satisfy QoS, the base station transfers the active session with $S_{L\bar{L}}$ to the network segment $NS_{\bar{L}}$. If in this case, the communication characteristics do not satisfy QoS, then the session with $S_{L\bar{L}}$ is interrupted by the base station. At the same time, note that the base station can forward an active session with $S_{L\bar{L}}$ to the network segment $NS_{\bar{L}}$ only if the subscriber device is within the coverage radius $r_{NS_{\bar{L}}}$ of this network segment, $r_{NS_{\bar{L}}}<r_{NS_{L}}$.

Parameterization of the service process of subscriber devices $S_{L\bar{L}}$ in network segment $NS_{L}$ is based on:

- distribution of the amount of communication resources necessary to support one session with $S_{L\bar{L}}$, taking into account QoS requirements,
- fraction of active sessions with $S_{L\bar{L}}$ that can be forwarded to network segment $NS_{\bar{L}}$.

To determine these characteristics, one should formalize the effective coverage radii $r_{NS_{L}}$, $r_{NS_{\bar{L}}}$ for network segments $NS_{L}$, $NS_{\bar{L}}$, respectively. We will analytically describe the process of determining the effective radius $r_{NS_{L}}=\min\left(r_{BS,BS},r_{BS,S}\right)$, where $r_{BS,BS}$ is the distance between base stations in the investigated 5G network, $r_{BS,S}$ is the coverage of the base station in the 5G network. We will define the radius $r_{BS,BS}$ as the maximum distance between the subscriber devices and the base station. Based on expression (3), we characterize the Signal-to-Noise Ratio (SNR) at a distance $r_{BS,BS}$ by the expression $R_{low}=K_{2}{\left(r_{BS,BS}^{2}+{\left(h_{BS}-h_{S}\right)}^{2}\right)}^{-\xi /2}$, where $K_{2}$ is the SNR corresponds to the lowest possible Modulation-and-Coding Scheme (MCS). Let's express the parameter $r_{BS,BS}$ from this equation: $r_{BS,BS}=\sqrt{{\left(\frac{R_{low}L_{R}}{K_{2}}\right)}^{2/\xi }-{\left(h_{BS}-h_{S}\right)}^{2}}$, where $L_{R}=\sqrt{2}\sigma_{L_{R}}/\text{erfc}\left(2p_{BS}\right)$ is the signal fading limit, erfc is the additional Gaussian error function, $\sigma_{L_{R}}$ is a signal fading limit standard deviation (see Ref. [36]), $p_{BS}$ is a cell boundary coverage probability. Note that usually $r_{BS,BS}<r_{BS,S}$ is due to differences in carrier frequencies and permissible radiation power. The radius $r_{BS,S}$ equal to half the distance between neighboring base stations is determined as a result of the approximation of the coverage circle by the Dirichlet division, in which we take into account the location of the base station at $R^{2}$. To determine the Dirichlet partition for $R^{2}$, we resort to mathematical modeling [39]. Thus, both components $r_{BS,BS}$, $r_{BS,S}$ needed to determine the effective radius $r_{NS_{L}}$ will be formalized. The procedure for determining the effective radius $r_{NS_{\bar{L}}}$ is methodologically similar to the one just described for $r_{NS_{L}}$.

Now let's estimate the amount of communication resources needed to support a session with $S_{L\bar{L}}$. The base element of such estimation is the SNR distribution function. The stochastic nature of the SNR in the investigated 5G network is determined by the variability of the location of the subscriber device relative to the base station and fading [36].

We will get an analytical estimate of the distance distribution function between the subscriber device and the base station in three-dimensional space. The distance on the plane is distributed according to the distribution density [40]: $w_{r}\left(y\right)=2y/r_{NS_{L}}^{2}$. According to the method of transformation of stochastic quantities [41]: if a stochastic quantity Χ with a distribution density w(x) is a function X=ψ(Y) of another stochastic quantity Y with a distribution density υ(y), then

(6) $$w\left(x\right)=\sum_{i}f\left(\gamma_{i}\left(x\right)\right)\left|\frac{d\gamma_{i}^{\prime }\left(x\right)}{dx}\right|\text{,}$$

where $y=\gamma_{i}\left(x\right)=1/\psi \left(y\right)$ are inverse functions.

Based on (6), for $w_{r}\left(y\right)$ and $\psi_{d}\left(r\right)=\sqrt{r^{2}+{\left(h_{BS}-h_{S}\right)}^{2}}$ we obtain an analytical expression for the SNR without fading: $W_{d}\left(y\right)=\left(2h_{BS}h_{S}-h_{BS}^{2}+h_{S}^{2}+y^{2}\right)/d^{2}$, where $h_{BS}-h_{S}<y<\sqrt{r_{NS_{L}}^{2}+h_{BS}^{2}-2h_{BS}h_{S}+h_{S}^{2}}$, and d is the distance between $S_{L\bar{L}}$ and the base station in three-dimensional space. We define the inverse function of $W_{d}\left(y\right)$: $\psi_{SNR,dB}\left(d\right)=10\;\mathrm{lg}\left(ad^{-\xi }\right)$, where а is a coefficient that summarizes all gains and losses (except propagation losses and fluctuations caused by fading); ξ is a coefficient that characterizes propagation losses. By substituting the functions $W_{d}\left(y\right)$ and $\psi_{SNR,dB}\left(d\right)$ in (6), we obtain the SNR distribution function $W_{SNR,dB}\left(y\right)=1-\frac{10^{-x/5\xi }a^{2/\xi }-{\left(h_{BS}-h_{S}\right)}^{2}}{r_{NS_{L}}^{2}}$ at $y>10\;\mathrm{lg}\left(a{\left({\left(h_{BS}-h_{S}\right)}^{2}+r_{NS_{L}}^{2}\right)}^{-\xi /2}\right)$.

Note that attenuation is typically estimated by a log-normal distribution on a linear scale. When switching to a normal distribution on the decibel scale, the stochastic value of the SNR with attenuation taken into account can be represented by the expression $W_{SNR,Att,dB}=W_{SNR,dB}+N\left(0,\sigma_{Att}\right)$, where $\sigma_{Att}$ there is a fading standard deviation. Let us define the SNR distribution function taking into account the fading as a result of convolution $W_{SNR,dB}\left(y\right)$ with a normal distribution with zero mean and variance

(7) $$\sigma_{Att}:W_{SNR,Att,dB}\left(y\right)=\int_{-\infty }^{\infty }\frac{\exp\left(-u^{2}/2\sigma_{Att}^{2}\right)}{\sqrt{2\pi }\sigma_{Att}}W_{SNR,dB}\left(y+u\right)du\text{.}$$

It is impossible to obtain a closed estimate of the last expression in terms of the transformation of stochastic quantities, so let's move on to the representation of (7) in terms of Laplace functions:

(8) $$W_{SNR,Att,dB}\left(y\right)=\frac{1}{2r_{NS_{L}}^{2}}(a^{2/\xi }10^{-y/5\xi }\;\exp\left(\left(\sigma_{Att}^{2}\;\log^{2}\;10\right)/\left(50\xi^{2}\right)\right)\times \times (\Phi (\left(50\xi \;\log\;a-25\xi^{2}\;\log\;b+\sigma_{Att}^{2}\;\log^{2}\;10-\frac{5\xi y\;\log\;10}{5\sqrt{2}\xi \sigma_{Att}\;\log\;10}\right)--\Phi \left(\left(50\xi \left(\log\;a-\xi \;\log\left(h_{BS}-h_{S}\right)\right)+\sigma_{Att}^{2}\;\log^{2}\;10-\frac{5\xi y\;\log\;10}{5\sqrt{2}\xi \sigma_{Att}\;\log\;10}\right)\right)++\left(r_{NS_{L}}+{\left(h_{BS}-h_{S}\right)}^{2}\right)\Phi \left(\frac{y\;\log\;10-10\;\log\;a+5\xi \;\log\;b}{\sqrt{2}\sigma_{Att}\;\log\;10}\right)--{\left(h_{BS}-h_{S}\right)}^{2}\Phi \left(\frac{\sqrt{2}\left(10\xi \;\log\left(h_{BS}-h_{S}\right)-10\;\log\;a\right)+y\;\log\;10}{\sigma_{Att}\;\log\;100}\right)+r_{NS_{L}}^{2})\text{,}$$

where Φ is the Laplace function, $b=r_{NS_{L}}^{2}+{\left(h_{BS}-h_{S}\right)}^{2}$.

Note that in function (8) it is possible to take into account the appearance of obstacle in the space between the subscriber device and the base station (in the value of the propagation coefficient а and by defining the dispersion $\sigma_{Att}$ as $\sigma_{Att}=\left\{\sigma_{Att,O},\sigma_{Att,\bar{o}}\right\}$, where $\sigma_{Att,O}$, $\sigma_{Att,\bar{O}}$ are options for determining the dispersion $\sigma_{Att}$ with and without taking into account the presence of obstacle, $\sigma_{Att,\bar{O}}=\sigma_{Att}$). Accordingly, it is possible to obtain two variants of the function (8): $W_{SNR,Att,O,dB}\left(y\right)$, $W_{SNR,Att,\bar{O},dB}\left(y\right)=W_{SNR,Att,dB}\left(y\right)$. Weighing these functions by the value of the probability of the occurrence of an obstacle $p_{O}$ (see expression (4)), we obtain a universal version of the expression for estimating the SNR distribution function:

(9) $$W_{SNR}\left(y\right)=p_{O}W_{SNR,Att,O,dB}\left(y\right)+\left(1-p_{O}\right)W_{SNR,Att,dB}\left(y\right)\text{.}$$

At the given Block Error Rate, the desired distribution function of the amount of communication resources required to support a subscriber device session in the investigated 5G network is obtained as a result of comparing the function (9) with the limit values of SNR for each MCS [42] supported by the base station. Due to the offset of the effective radii $r_{NS_{L}}$, $r_{NS_{\bar{L}}}$, the actual costs of switching resources to maintain a session of the target subscriber device $S_{L\bar{L}}$ can vary greatly depending on which network segment ($NS_{L}$ or $NS_{\bar{L}}$) it connects to. We assume that the subscriber devices $S_{L\bar{L}}$ are distributed over $R^{2}$ according to a point Poisson process.

Considering the value of the MCS spectral efficiency, let's express the average spectral efficiency of the session in the investigated 5G network as $E\left(R_{l}\right)=\frac{2}{r_{NS_{L}}}\int_{0}^{r_{NS_{L}}}y\;\log_{2}\left(1+R\left(y\right)\right)dy$, where R(y) is the SINR for the subscriber device $S_{L\bar{L}}$ located at a distance l from the base station (see expression (3)).

### 2.4 The concept of 5G network operation in licensed and unlicensed frequency ranges

In Section 2.1, we determined that the information space of the investigated 5G network includes network segments $NS_{L}$, $NS_{\bar{L}}$ deployed in licensed and unlicensed frequency spaces, respectively. In Section 2.3, we analytically justified the corresponding effective radii of the coverage $r_{NS_{L}}$, $r_{NS_{\bar{L}}}$ for $NS_{L}$, $NS_{\bar{L}}$, moreover, $r_{NS_{L}}>r_{NS_{\bar{L}}}$. Also, note that QoS requirements are applied to sessions supported in the network segment $NS_{L}$ (unlike sessions supported in the network segment $NS_{\bar{L}}$).

In this context, let's define two types of sessions characteristic of the investigated 5G network. Sessions of the first type, characteristic of subscriber devices $S_{L\bar{L}}$ located at a distance $r_{NS_{L}}>y>r_{NS_{\bar{L}}}$ from the base station, can be served exclusively in the network segment $NS_{L}$ (if the base station has enough communication resources to meet QoS requirements). If the QoS requirements are not met, then the base station cannot physically redirect the session of the first type to the network segment $NS_{\bar{L}}$, i.e. the corresponding subscriber device is outside the latter's coverage area. Typical for subscriber devices $S_{L\bar{L}}$ located at a distance $y\leq r_{NS_{\bar{L}}}$ from the base station, sessions of the second type are initially accepted for service in the network segment $NS_{L}$. If the QoS requirements are not met for the session of the second type in $NS_{L}$, then the base station forwards such a session to the network segment $NS_{\bar{L}}$.

Let us define the intensity of session activation requests in the investigated 5G network as $\eta =\mu_{L\bar{L}}\pi r_{NS_{L}}^{2}p_{T}\eta_{S_{L\bar{L}}}=\eta_{1}+\eta_{2}$, where $\mu_{L\bar{L}}$ is the density of the location of subscriber devices per square meter in $R^{2}$ (see Section 2.1), $\pi r_{NS_{L}}^{2}$ is the coverage circle for the network segment $NS_{L}$ (see Section 2.3), $p_{T}$ is the probability that the session with $S_{L\bar{L}}$ is active, $\eta_{S_{L\bar{L}}}$ is the intensity with which $S_{L\bar{L}}$ generates requests for session activation, $\eta_{1}$ is the intensity of generating requests for activating sessions of the first type, $\eta_{2}$ is the intensity of generating requests for activating sessions of the second type. Accordingly, we define the intensity $\eta_{L}$ of incoming requests to the network segment $NS_{L}$ as $\eta_{L}=\eta$. We define the intensity $\eta_{\bar{L}}$ of incoming requests to network segment $NS_{\bar{L}}$ as $\eta_{\bar{L}}=\eta_{2}q_{2}$, where $q_{2}$ is the probability of forwarding a session of the second type to network segment $NS_{\bar{L}}$. In turn, the probability of losing a session of the first type is denoted by $q_{1}$.

The target quality indicator that will characterize the efficiency of the studied 5G network will be the required density of base stations placed in the area $R^{2}$, necessary to guarantee the minimum declared QoS speed $V_{\min}$ for the given densities $\mu_{L\bar{L}}$, $\mu_{\bar{L}}$ of the presence of subscriber devices $S_{L\bar{L}}$, $S_{\bar{L}}$ in this area, respectively.

Modeling of the process of session maintenance in the network segment $NS_{L}$ is carried out based on the queuing theory. More specifically, let's examine the queuing system with the amount of communication resources $A_{R}$, which describes the process of servicing $N_{S}$ sessions, where $A_{R},N_{S}\in R$, $N_{S}$ represents the number of serviced subscriber devices $S_{L\bar{L}}$: $N_{S}=N_{S_{L\bar{L}}}$. Two streams of requests to activate sessions of the first and second types with intensities $\eta_{1}$, $\eta_{2}$, respectively, are received at the system input.

The service duration is exponentially distributed with parameters μ. To service the session, the system should use the amount of communication resources equal to $a_{R}$, $0<a_{R}\leq A_{R}$. We regulate the resource consumption distribution of requests depending on the types of sessions in the form of a set ${\left\{p_{s,a}\right\}}_{a\geq 0}$, where $p_{s,a}$ is the probability that the amount of communication resources equal to а is required to support a session of type s={1,2}.

According to Ref. [43], let's move on to the analysis of the queuing system with one aggregated flow of incoming requests instead of the two described above. At the same time, we define the resource requirements in such an aggregated flow as $p_{a,L}=\left(\beta_{1}p_{1,a}+\beta_{2}p_{2,a}\right)/\beta$, where $\beta_{s}=\eta_{s}/\mu$, $\beta =\sum_{s}\beta_{s}$, s={1,2}. The request for session activation is satisfied if there are enough free communication resources in the system at the time of its receipt. Otherwise: if it is a request to activate a session of the first type, then the request is rejected, if it is a request to activate a session of the second type, then the request is forwarded to the network segment $NS_{\bar{L}}$. In any case, the service time of an active session is limited. In this context, the behavior of the system will be described by the stochastic process $X\left(t\right)=\left(N_{S}\left(t\right),A_{R}\left(t\right)\right)$, where $N_{S}\left(t\right)$ is the number of sessions supported by the system; $A_{R}\left(t\right)=\left\{a_{i}\left(t\right)\right\}$, $i=\bar{1,N_{S}}$, is the number of communication resources reserved for the service of the і th active session at a time t. Let us define as $P_{s}\left(a_{R}\right)$ the stationary probability that the system serves s sessions, for which the amount of communication resources equal to $a_{R}$ is spent: $P_{s}\left(a_{R}\right)=\lim_{t\to \infty }\left\{N_{S}\left(t\right)=s,\sum_{i=1}^{s}a_{i}\left(t\right)=a_{R}\right\}$, $0<a_{R}\leq A_{R}$. For $s=\bar{1,N_{S}}$, $a_{R}=\bar{1,A_{R}}$, we represent the expression for $P_{s}\left(a_{R}\right)$ as

(10) $$P_{s}\left(a_{R}\right)=\left(P_{0}\beta^{s}p_{a_{R},L}^{s}\right)/s!\text{,}$$

where $P_{0}=1/\left(1+\sum_{s=1}^{N_{S}}\frac{\beta^{s}}{s!}\sum_{a_{R}=1}^{A_{R}}p_{a_{R},L}^{\left(s\right)}\right)$; $p_{a_{R},L}^{\left(s\right)}$ is a recursively computed convolution operation of the ${\left\{p_{a_{R},L}\right\}}_{a_{R}>0}$ distribution:

$$p_{a_{R},L}^{\left(s\right)}\sum_{i=1}^{a_{R}}p_{a_{R}-i,L}^{\left(s-1\right)}p_{i,L}\text{.}$$

In the context of (10), the probability $q_{2}$ of losing a session of the second type is defined as

(11) $$q_{2}=1-P_{0}\sum_{s=0}^{N_{S}-1}\frac{\beta^{s}}{s!}\sum_{a_{R}=0}^{A_{R}}p_{a_{R},L}^{\left(s+1\right)}\text{.}$$

Note the computational complexity of the direct calculation of the characteristic $q_{2}$ according to expression (11) at large values of $N_{S}$, $A_{R}$. Accordingly, we propose a simplified convolution procedure for calculating $q_{2}$:

$$q_{2}=1-\left(\sum_{a_{R}=0}^{A_{R}}p_{2,a_{R}}H\left(N_{S}-1,A_{R}-a_{R}\right)\right)/G\left(N_{S},A_{R}\right)\text{,}$$

where the recursively computed function $H\left(N_{S},a_{R}\right)$ is interpreted as $H\left(N_{S},a_{R}\right)=\sum_{s=0}^{N_{S}}\frac{\beta^{s}}{s!}\sum_{i=0}^{a_{R}}p_{i,L}^{\left(i\right)}$.

On the same basis, we determine the probability $q_{1}$ of losing a session of the first type:

$$q_{1}=1-\left(\sum_{a_{R}=0}^{A_{R}}p_{1,a_{R}}H\left(N_{S}-1,A_{R}-a_{R}\right)\right)/H\left(N_{S},A_{R}\right)\text{.}$$

Now let's describe the process of servicing $S_{L\bar{L}}$-sessions forwarded to the network segment $NS_{\bar{L}}$. The distribution of requirements for the amount of resources necessary to support sessions redirected to the network segment $NS_{\bar{L}}$ is expressed as

(12) $$p_{i,\bar{L}}=p_{2,i}\left(\sum_{a_{R}=0}^{A_{R}}P_{N_{S}}\left(a_{R}\right)+\sum_{s=0}^{N_{S}-1}\sum_{a_{R}=A_{R}-i+1}^{A_{R}}P_{s}\left(a_{R}\right)\right)/q_{2}\text{,}$$

where $0\leq i\leq A_{R}$, the characteristic $P_{s}\left(a_{R}\right)$ is described by expression (10), and the probability $q_{2}$ is determined by expression (11). Distribution (12) can be expressed in terms of the function $H\left(s,a_{R}\right)$:

$$p_{i,\bar{L}}=p_{2,i}\left(H\left(N_{S},A_{R}\right)-H\left(N_{S}-1,A_{R}-i\right)\right)/\left(q_{2}H\left(N_{S},A_{R}\right)\right)\text{.}$$

Sessions from network segment $NS_{L}$ (activated by subscriber devices $S_{L\bar{L}}$) redirected to network segment $NS_{\bar{L}}$ compete for resources with requests from subscriber devices $S_{\bar{L}}$ that are definitively addressed to network segment $NS_{\bar{L}}$. We take this into account when determining the probability $p_{ST}$ of successful transmission for devices $S_{L\bar{L}}$, $S_{\bar{L}}$, taking into account both the probability $p_{D}$ of distortions caused by the simultaneous operation of devices $S_{L\bar{L}}$, $S_{\bar{L}}$, and the probability $p_{O}$ of obstacle appearance in the space between the subscriber device and the base station:

(13) $$p_{ST}=\left(1-p_{D}\right)\left(1-p_{O}\right)\text{.}$$

**Fig. 1:**
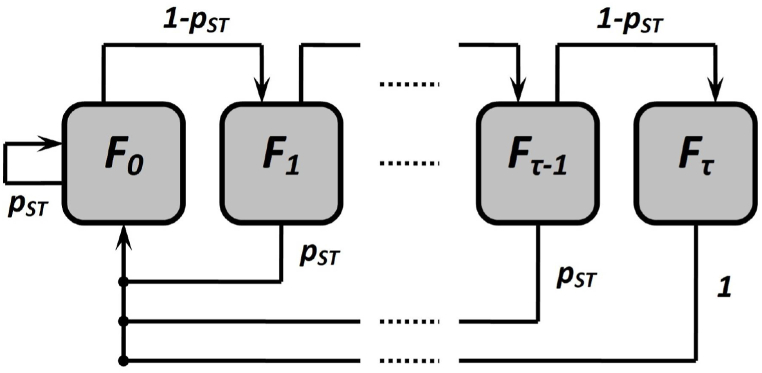
UML state diagram for Markov chain F.

Section 2.1 was devoted to the specifics of the organization of communication of subscriber devices with the base station in the context of the phenomena that determine the value of probabilities $p_{D}$, $p_{O}$. Let us present the corresponding procedure for random access to the transmission medium in the form of a Markov chain $F=\left\{F_{n},n\geq 0\right\}$, where the parameter $F_{n}$ represents the number of unsuccessful data transmission attempts by the subscriber device since the last successful attempt, $F_{n}\in \left[0,\tau \right]$. The graphical interpretation of the Markov chain in the form of a UML state diagram is presented in Fig. 1.

We formalize the system of equations for the stationary distribution F in terms of probabilities ρ of realization of the corresponding states of this Markov process:

(14) $$\left\{ \begin{array}{l} \rho_{0}=\rho_{0}p_{ST}+\rho_{1}p_{ST}+\ldots +\rho_{\tau -1}p_{ST}+\rho_{\tau },\\ \rho_{i}=\rho_{i-1}\left(1-p_{ST}\right)\forall i=\bar{1,\tau -1},\\ \rho_{\tau }=\rho_{\tau -1}\left(1-p_{ST}\right). \end{array}\right.\text{.}$$

Having analytically solved the system (14), we present the stationary probabilities $\rho_{і}$, $i=\bar{0,\tau }$, as

(15) $$\rho_{i}=\left(p_{ST}{\left(1-p_{ST}\right)}^{i}\right)/\left(1-{\left(1-p_{ST}\right)}^{\tau +1}\right)\text{,}$$

We denote by $q_{L\bar{L}}$, $q_{\bar{L}}$ the probabilities that subscriber devices $S_{L\bar{L}}$, $S_{\bar{L}}$, respectively, make requests to activate sessions at the same time. Considering the existence of competing sessions $n_{L\bar{L}}$, $n_{\bar{L}}$, the probability $p_{D}$ is expressed as

(16) $$p_{D}=\left(1-p_{O}\right)\left(1-{\left(1-q_{L\bar{L}}\right)}^{n_{L\bar{L}}}{\left(1-q_{\bar{L}}\right)}^{n_{\bar{L}}}\right)\text{,}$$

where the probability $p_{O}$ is determined by expression (4).

Since subscriber devices $S_{L\bar{L}}$, $S_{\bar{L}}$ try to transmit data in one tact of each state $\left\{F_{n}\right\}$, the probability $q_{L\bar{L}}$ can be interpreted as the inverse of the average number of tacts the Markov chain stays in the unchanged state:

(17) $$q_{L\bar{L}}=1/\sum_{i=0}^{\tau }\rho_{j}z_{j}\text{,}$$

where $z_{j}$ is the average number of tacts of $\left\{F_{n}\right\}$ being in state j:

(18) $$z_{j}=\sum_{i=1}^{2^{j}S_{CW}}\frac{i}{2^{j}S_{CW}}=\frac{1}{2}\left(2^{j}S_{CW}+1\right)\text{,}$$

where $j=\bar{0,\tau }$; $S_{CW}$ is the initial size of the Contention Window (see Section 2.1).

Let's redefine expression (17) by taking into account expressions (15), (18):

(19) $$q_{L\bar{L}}=1/\left(\frac{p_{ST}S_{CW}\left(1-2^{\tau +1}\right)}{2\left(1-{\left(1-p_{ST}\right)}^{\tau +1}\left(2p_{ST}-1\right)\right)}+\frac{1}{2}\right)\text{.}$$

The expression for calculating $q_{\bar{L}}$ is identical to expression (19): $q_{\bar{L}}\equiv q_{L\bar{L}}$.

It should also be taken into account that the stochastic characteristics of $p_{ST}$, $p_{D}$, $q_{L\bar{L}}$, $q_{\bar{L}}$ actually depend on the number of competing sessions $n_{L\bar{L}}$, $n_{\bar{L}}$. Therefore, after solving the nonlinear system (13), (16), (19) for each pair of $n_{L\bar{L}}\in$, $n_{\bar{L}}$ values, the obtained results can be used to determine the resulting probability $P_{L\bar{L}}$ of data transmission for $S_{L\bar{L}}$ in the investigated 5G network:

(20) $$P_{L\bar{L}}=\sum_{i=1}^{\infty }\frac{{\left(\beta_{L\bar{L}}^{*}\right)}^{i}}{i!}\exp\left(-\beta_{L\bar{L}}^{*}\right)\sum_{j=0}^{\infty }\sum_{i=1}^{\infty }\frac{{\left(\beta_{\bar{L}}^{*}\right)}^{j}}{j!}\exp\left(-\beta_{\bar{L}}^{*}\right)q_{\bar{L}}\left(i,j\right)p_{ST}\left(i,j\right)\text{,}$$

where $\beta_{L\bar{L}}^{*}=\eta_{\bar{L}}/\mu$, $\beta_{\bar{L}}^{*}={\eta_{\bar{L}}/\mu }_{\bar{L}}$. The resulting probability $P_{\bar{L}}$ for $S_{\bar{L}}$ is calculated similarly.

Since the speed of a session with spectral efficiency $e_{j}$ is determined by the expression $V_{\bar{L},j}^{L\bar{L}}=P_{L\bar{L}}W_{\bar{L}}e_{j}$, where $W_{\bar{L}}$ is the channel width for the network segment $NS_{\bar{L}}$ (see the beginning of Section 2.1), the average speed for the $S_{L\bar{L}}$-sessions supported in the network segment $NS_{\bar{L}}$ is defined as

(21) $$E\left(V_{\bar{L}}^{L\bar{L}}\right)=\sum_{j=0}^{V}p_{j,\bar{L}}P_{L\bar{L}}W_{\bar{L}}e_{j}$$

(the expression for $S_{\bar{L}}$-sessions is similar). The probability $p_{j,\bar{L}}$ ∀j is calculated by expression (12).

Finally, it remains for us to characterize the resulting probability Q of request consumption in the investigated 5G network. We define this indicator by the probability of an event when, for a $S_{L\bar{L}}$-session forwarded to the network segment $NS_{\bar{L}}$, the minimum actual speed V turns out to be less than the value $V_{\min}$ specified in QoS: $Q_{\bar{L}}=P\left\{V<V_{\min}\right\}=\sum_{V_{\bar{L},j}^{L\bar{L}}<V_{\min}}p_{j,\bar{L}}$. Therefore, we define the characteristic Q as

(22) $$Q=\left(q_{1}\left(\eta_{1}+\eta_{2}\left(1-q_{2}\right)\right)+Q_{\bar{L}}\eta_{2}q_{2}\right)/\eta \text{.}$$

**Fig. 2:**
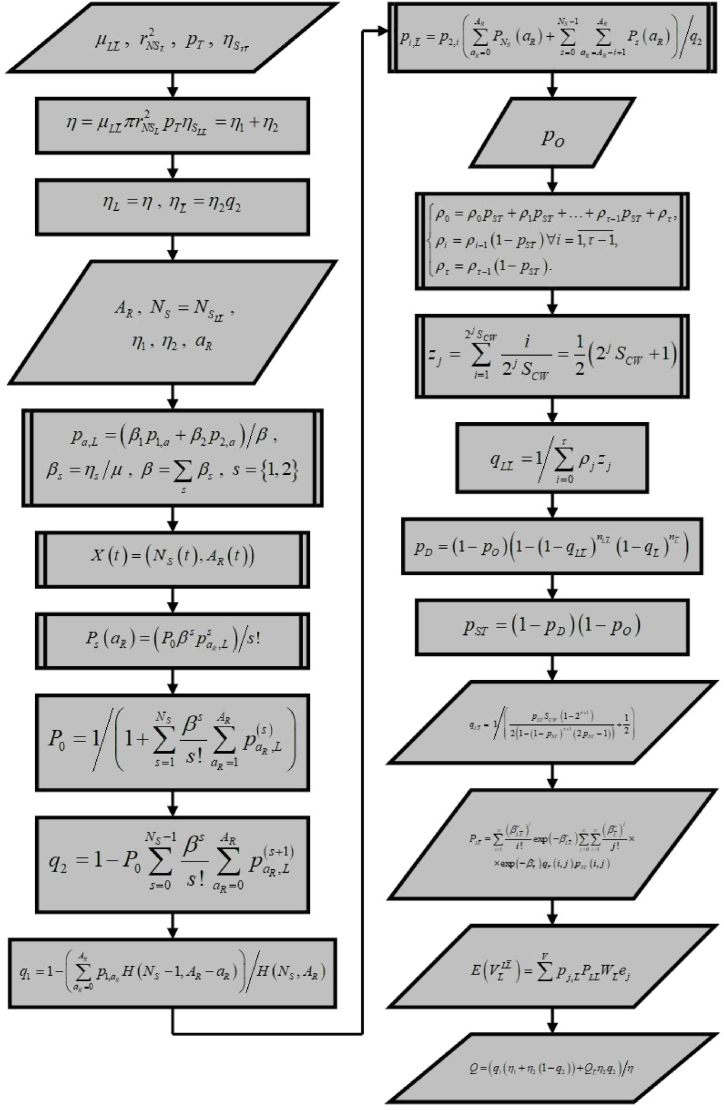
A flow chart diagram for the proposed concept of 5G network operation in licensed and unlicensed frequency ranges.

Below we summarize the formalization of the proposed concept of 5G network operation in licensed and unlicensed frequency ranges in the form of a flow chart diagram, as shown in Fig. 2.

The flow chart diagram presented in Fig. 2 demonstrates the order of computation for the parameters introduced in Section 2, which are necessary to characterize the quality of service in a 5G NRU network deployed in a target area characterized by a specified subscriber density. Some parameters shown in the diagram are computed cyclically. To accurately interpret the elements of Fig. 2, it is essential to refer to the corresponding fragments of the material in Section 2.

## 3 Results

We will apply the mathematical apparatus presented in Section 2 for simulation modeling of the investigated 5G network. As a reliable simulation environment, we will use MathWorks MATLAB R2022a with a 5G Toolbox installed. The latter contains not only standardized waveforms but also a toolkit for modeling and verifying communication systems such as 3GPP 5G New Radio (Release 15), Wi-Fi, Zigbee, and NFC. MATLAB also allows us to apply the Monte Carlo technique to validate the values of the calculated metrics for more than 100 variations of channel implementations.

Among the main established parameters that were used to calculate the following metrics, we note: operating frequency: $f_{L}=28$ GHz, $f_{\bar{L}}=38$ GHz; channel width: $W_{L}=200$ MHz, $W_{\bar{L}}=160$ MHz; base station placement height $h_{BS}=10$ m; parameters of a pedestrian who can become an obstacle in the space between the subscriber device and the base station: height $h_{P}=2$ m, radius $r_{P}=0.25$ m, the intensity of appearance $\eta_{P}=0.33$; the height of placement of subscriber device $h_{S}=1.5$ m; transmitter power: $P_{BS,L}=33$ dBm, $P_{BS,\bar{L}}=23$ dBm; thermal noise $N_{0}=-174$ dBpHz; interference immunity margin $L_{I}=5$ dB; signal reception power threshold $S_{low}=-9$ dB; base station antenna array configuration for $NS_{L}$: 64×4; base station antenna array configuration for $NS_{\bar{L}}$: 16×4; antenna array configuration of subscriber devices $S_{L\bar{L}}$, $S_{\bar{L}}$: 8×4; activation probability of $S_{L\bar{L}}$-session $\beta_{L\bar{L}}=0.1$; activation probability of $S_{\bar{L}}$-session $\beta_{\bar{L}}=0.1$; initial value for Contention Window $S_{CW}$: from 8; the number of retransmission attempts with doubling of Contention Window $N_{S_{CW}}$: from 10; inserted QoS for $S_{L\bar{L}}$ minimum session speed $V_{\min}=50$ Mbps.

A mandatory element of mathematical modeling of the researched process is proving the adequacy of the obtained model for a defined range of characteristic parameters. Of course, we cannot bypass this stage of responsibilities. At the same time, we note an objective complication: testing a 5G network in an unlicensed frequency range in the conditions of urban infrastructure is illegal. So let's focus on the licensed frequency range.

**Fig. 3:**
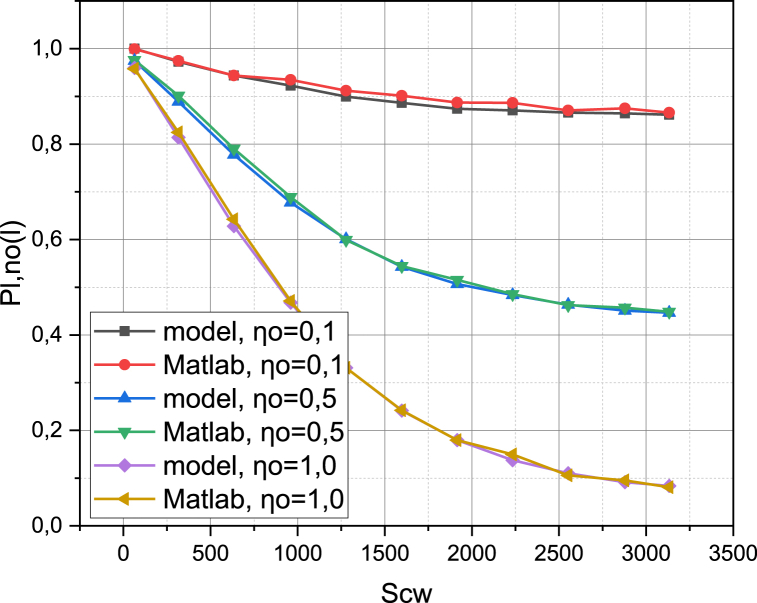
Visualization of functional dependence $P_{L\bar{L}}\left\{Matlab,model\right\}=f\left(S_{CW},\eta_{O}\right)$.

We compare the metric of the probability $P_{L\bar{L}}$ of successful data transmission for $S_{L\bar{L}}$ as a result of the operation of the real investigated 5G network (real, the metric $P_{L\bar{L}}$ is calculated as a share of the amount of successfully transmitted data in the total amount of data prepared for transmission) and the model of the investigated 5G network (model, the metric $P_{L\bar{L}}$ is calculated according to expression (20)). Arguments in determining the functional dependence $P_{L\bar{L}}$ will be the value for the Contention Window $S_{CW}$ and the discrete values of the intensity of the appearance of a pedestrian obstacle in the space between the subscriber device and the base station $\eta_{O}=\left\{0.1,0.5,1.0\right\}$. Among other established parameters, note $\beta_{L\bar{L}}=0.1$ and the configuration of the antenna array of subscriber devices $S_{L\bar{L}}$: 8×4. The resulting graphs of dependence $P_{L\bar{L}}\left\{Matlab,model\right\}=f\left(S_{CW},\eta_{O}\right)$ are presented in Fig. 3. These graphs are a generalization of a statically reliable number of results of controlled experiments.

Presented in Fig. 1 dependences are close enough for all values of the argument and quantities $\eta_{O}$. This empirically proves the adequacy of the mathematical apparatus presented in Section 2 for describing the process of functioning of the investigated 5G network in the licensed frequency range. We obtain the results of the operation of the research object in the unlicensed frequency range by implementing the mathematical models presented in Section 2 in the verified MathWorks MATLAB environment (5G Toolbox), which allows us to draw an inductive conclusion about their adequacy.

Now let's use the mathematical apparatus presented in Section 2 to study various aspects of the functioning of a 5G network with active network segments $NS_{L}$, $NS_{\bar{L}}$.

**Fig. 4:**
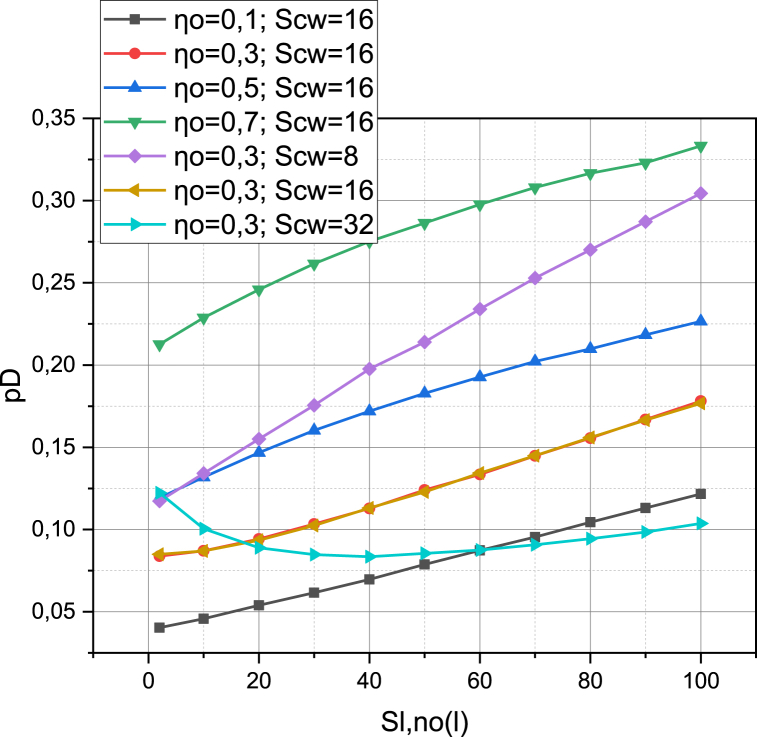
Visualization of functional dependence $p_{D}=f\left(N_{S_{L\bar{L}}},\eta_{O},S_{CW}\right)$.

Let's investigate how probable the situation in which subscriber devices $S_{L\bar{L}}$, $S_{\bar{L}}$ make requests to activate sessions at the same time. As mentioned, this situation can be a technological cause of transmission failure (several subscriber devices appear in the coverage sector of the directional antenna array of the base station, which simultaneously initiates transmission processes). The metric in this study will be the probability $p_{D}$ characterized by expression (16). The arguments will be the number $N_{S_{L\bar{L}}}$ of simultaneously working subscriber devices $S_{L\bar{L}}$ on $R^{2}$, the intensity of obstacle appearance $\eta_{O}=\left\{0.1,0.3,0.5,0.7\right\}$ and the value for the Contention Window $S_{CW}=\left\{8,16,32\right\}$. The calculated dependencies $p_{D}=f\left(N_{S_{L\bar{L}}},\eta_{O},S_{CW}\right)$ are presented in Fig. 4.

**Fig. 5:**
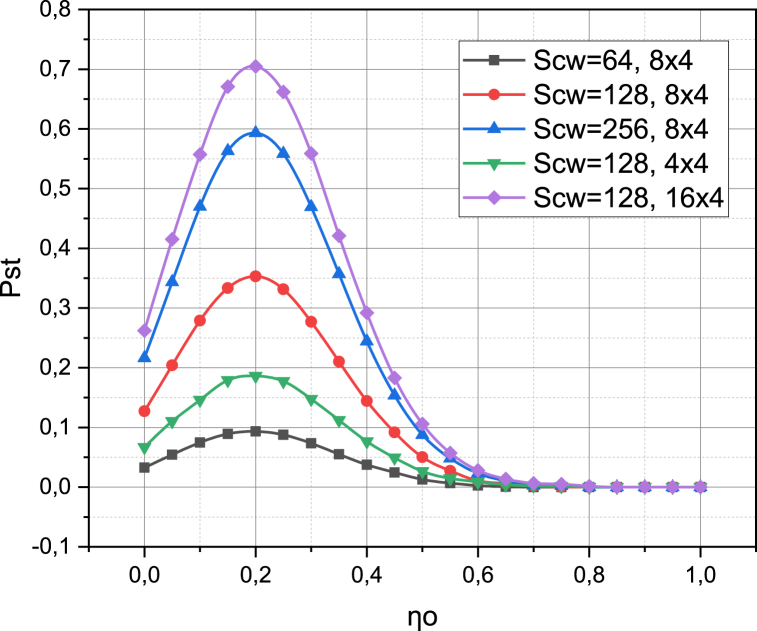
Visualization of functional dependence $p_{ST}=f\left(\eta_{O},S_{CW},arr\_conf\right)$.

Now let's investigate how likely it is that a transmission failure occurs due to a physical reason in the investigated 5G network (n obstacle opaque to radio radiation, in particular, a person, appeared in the space between the subscriber device and the base station). We will conduct the research in the probability metric $p_{ST}$ for $S_{L\bar{L}}$, $S_{\bar{L}}$ (see expression (13) with an emphasis on expression (16) and (4)). The arguments will be the intensity $\eta_{O}$ of the obstacle appearance on a square meter of the target area $R^{2}$, the configuration of the antenna array of the subscriber device {4×4,8×4,16×4}, and the value for Contention Window $S_{CW}=\left\{64,128,256\right\}$. The calculated dependencies $p_{ST}=f\left(\eta_{O},S_{CW},arr\_conf\right)$ with a share of $S_{L\bar{L}}$ sessions in system $\beta_{\bar{L}}=0.2$ are presented in Fig. 5.

**Fig. 6:**
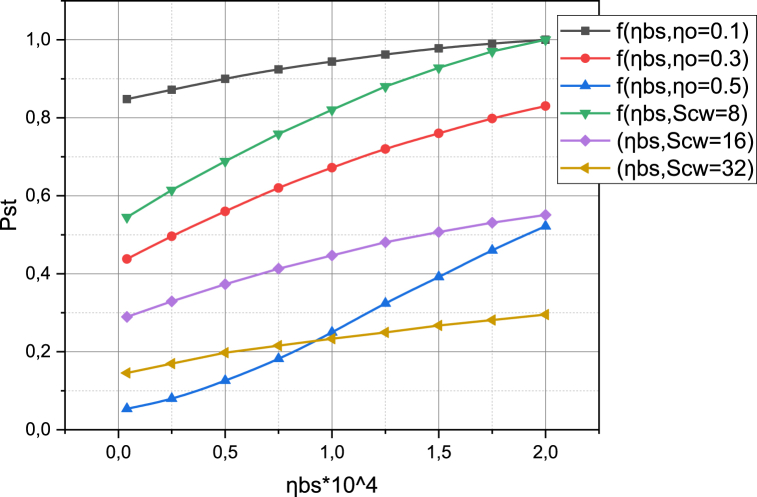
Visualization of functional dependence $p_{ST}=f\left(\eta_{BS},S_{CW}\right)$.

Now let's focus on estimating the probability $p_{ST}$ of successful transmission in both network segments $NS_{L\bar{L}}$, $NS_{\bar{L}}$ of the investigated 5G network as a function of the density $\eta_{BS}$ of base station placement on the target area $R^{2}$. At the same time, we take into account that the characteristics $\eta_{O}$ $S_{CW}$ can take values from the sets {0.1,0.3,0.5}, {8,16,32}, respectively. The calculated values for the dependences of $p_{ST}=f\left(\eta_{BS},\eta_{O}\right)$, $p_{ST}=f\left(\eta_{BS},S_{CW}\right)$ are presented in the form of graphs in Fig. 6.

**Fig. 7:**
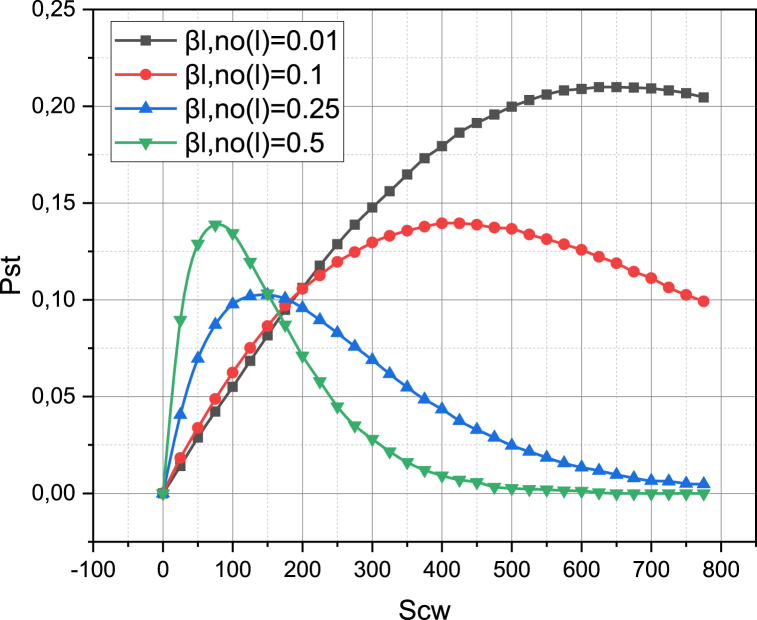
Visualization of functional dependence $p_{ST}=f\left(S_{CW},\beta_{L\bar{L}}\right)$.

Let's complete the study of the metric $p_{ST}$ by defining it for the case when all the traffic of the investigated 5G network will be directed to the network segment $NS_{\bar{L}}$, which is deployed in an unlicensed frequency range. Recall that we can vary the share of sessions supported in network segments $NS_{L\bar{L}}$, $NS_{\bar{L}}$ by changing the value of parameters $\beta_{L\bar{L}}$, $\beta_{\bar{L}}$, respectively, ($\beta_{L\bar{L}}+\beta_{\bar{L}}=1$, $\beta_{L\bar{L}},\beta_{\bar{L}}>0$). The results of calculating the dependence of $p_{ST}=f\left(S_{CW},\beta_{L\bar{L}}\right)$ at $\eta_{O}=0.7$ are presented in Fig. 7.

**Fig. 8:**
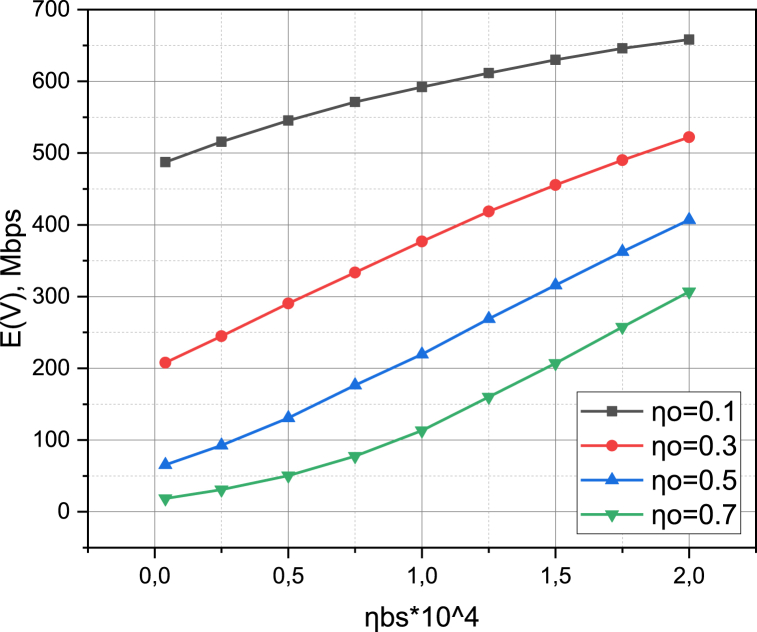
Visualization of functional dependence $E\left(V\right)=f\left(\eta_{BS},\eta_{O}\right)$.

**Fig. 9:**
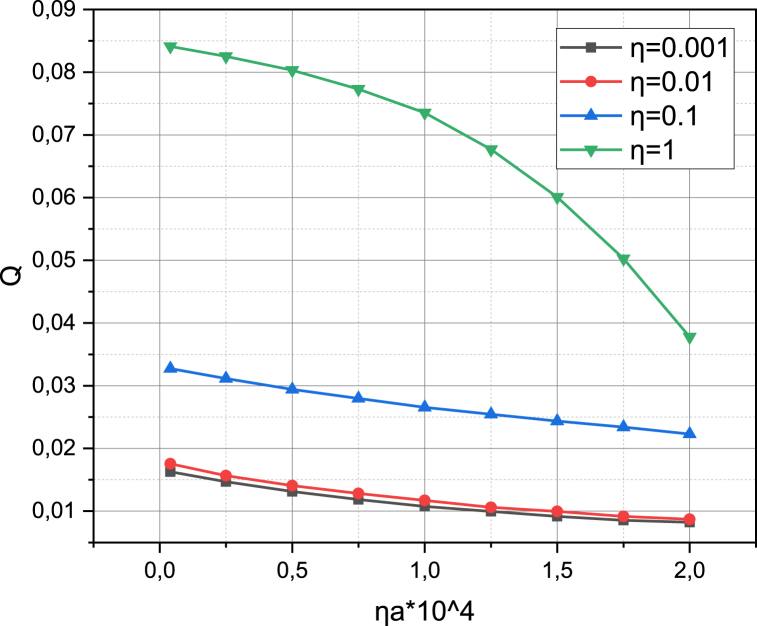
Visualization of functional dependence $Q=f\left(\eta_{BS},\eta \right)$.

Finally, let's characterize the investigated 5G network by calculating the functional dependence of the average speed $E\left(V_{\bar{L}}^{L\bar{L}}\right)=E\left(V\right)$ of data transmission (see expression (21)) and the total probability Q of the loss of requests (see expression (22)) on the density $\eta_{BS}$ of placing base stations on the target area $R^{2}$. When determining the dependence $E\left(V\right)=f\left(\eta_{BS}\right)$, we take into account that the intensity of the obstacle appearance $\eta_{O}$ took a value from the set {0.1,0.3,0.5,0.7}. We calculate the dependence $Q=f\left(\eta_{BS}\right)$ for the value of the parameter η={1,0.1,0.01,0.001}. The results of the calculation of $E\left(V\right)=f\left(\eta_{BS},\eta_{O}\right)$, $Q=f\left(\eta_{BS},\eta \right)$ are presented in Fig. 8, Fig. 9, respectively.

## 4 Discussion

We will conduct a sequential analysis of the results presented in Fig. 4, Fig. 5, Fig. 6, Fig. 7, Fig. 8, Fig. 9.

Fig. 4 represents the dependence of the probability of a transmission failure due to a technological reason (when several subscriber devices appear in the coverage sector of the directional antenna array of the base station and initiate transmission processes at the same time) on the number of such devices. All graphs of this dependence show an increasing trend with an increase in the value of the argument, which is expected. Interestingly, the values of the parameters $\eta_{O}$ (the density of the obstacle appearance intensity) and $S_{CW}$ (the contention window value) have a significant impact on the probability value $p_{D}$. For example, increasing the value of $\eta_{O}$ from 0.1 to 0.7 led to an overgrowth of the probability of $p_{D}$ by more than four times for all values of the argument (mostly, it is pedestrian obstacles that are simultaneously carriers of subscriber devices). We also note the linear character of the functions $p_{D}=f\left(N_{S_{L\bar{L}}},\eta_{O}\right)$. The reason for this is that, although the number of subscriber devices that have data to transmit and generate session activation requests increases as the probability $p_{D}$ increases, the Channel Observation-Based mechanism in operation controls the value of $S_{CW}$ effectively enough to contain the potential nonlinearity of the growth of the $p_{D}=f\left(N_{S_{L\bar{L}}},\eta_{O}\right)$ graphs. Confirmation of this conclusion can be seen by analyzing graphs of dependence $p_{D}=f\left(N_{S_{L\bar{L}}},S_{CW}\right)$. Having fixed the value of $S_{CW}=32$ in the range of values of $S_{L\bar{L}}\in \left[2,30\right]$, we see a non-linear character in the dependence of $p_{D}=f\left(N_{S_{L\bar{L}}},S_{CW}\right)$. In general, it can be predicted that there is a complex relationship between the parameters $\left\{p_{O},p_{D}\right\}$ and $\left\{\eta_{O},S_{CW}\right\}$.

The results presented in Fig. 5 develop the research just analyzed, because the probability $p_{ST}$ directly depends on the probabilities $p_{O}$, $p_{D}$ (see expression (13)). The nonlinear nature of the obtained dependencies fully demonstrates the sensitivity of the Listen-Before-Transmit technique used both to the hardware characteristics of the 5G network components (antenna array configuration) and to the intensity of information exchange in the latter and the degree of saturation of the space covered by obstacles. Note that increasing the complexity of antenna arrays has a positive effect on the value of the probability $p_{ST}$ (from the maximum $p_{ST}=0.2$ for the 4×4 configuration to $p_{ST}=0.7$ for the 16×4 configuration s). An increase in the value of the Contention Window $S_{CW}$ also has a positive effect on the value of the probability $p_{ST}$ (from the maximum of $p_{ST}=0.1$ at $S_{CW}=64$ to the maximum of $p_{ST}=0.6$ at $S_{CW}=256$). The most interesting result is that varying the values of $S_{CW}$, arr_conf changed the value, but not the position of the extremum of $p_{ST}$, which was always observed at the value of the argument $\eta_{O}=0.2$. Recall that this experiment was conducted for a fixed number of base stations and subscriber devices interacting in the ecosystem of the investigated 5G network. The parameter $\eta_{O}$ characterizes the intensity of obstacle appearance in the space between the base station and the subscriber device. The fixed position of the extremum $p_{ST}$ allows us to state that it is possible to set the problem of determining the number of base stations necessary for optimal service of a corresponding number of subscriber devices under the conditions of a given intensity of obstacle appearance in the target area. Such an optimization problem is particularly relevant at the stage of designing a 5G network to cover an area of $R^{2}$.

Taking into account the circumstances of the relationship between the base station location density and the density of subscriber devices accessibility per $R^{2}$, presented in Fig. 6 results are calculated precisely in the space of these generalized characteristics. At the same time, the base station location density varies in the range [0.2,2.0]. Additional factors are that the intensity of obstacle appearance varies in the range [0.1,0.5] (which covers the above-defined "critical value" of 0.2), and the parameter $S_{CW}$ takes on values from the set {8,16,32} (t a fixed value of $\eta_{O}=0.3$). It can be seen from the presented graphs that an increase in the base station's location density on $R^{2}$ has a positive effect on the probability of successful transmission of $p_{ST}$ for all values of $\eta_{O}$, $S_{CW}$. However, the nature of the graph's growth is linear and slow. This may indicate that the experiment was conducted for a relatively low density of location of subscriber devices per unit area $R^{2}$.

Fig. 7 shows the dependence of the probability of successful transmission $p_{ST}$ on the value of the parameter $S_{CW}$, taking into account the share of licensed traffic $\beta_{L\bar{L}}$ in the total traffic of the investigated 5G network. Note that the value of $S_{CW}$ has a very noticeable effect on the value of $p_{ST}$ for all variants of $\beta_{L\bar{L}}$. All graphs of dependency $p_{ST}=f\left(S_{CW},\beta_{L\bar{L}}\right)$ have a complex non-linear nature. These results are obtained for a high density of the obstacle appearance intensity $\eta_{O}=0.7$, so the narrower coverage radius of the unlicensed high-frequency range of the network segment $NS_{\bar{L}}$ gives an advantage, which is demonstrated by the curve $p_{ST}=f\left(S_{CW},\beta_{L\bar{L}}=0.01\right)$ (especially for $S_{CW}$ around 600). Therefore, it is precisely in conditions of the high density of the obstacle appearance intensity that the use of an unlicensed frequency range gives a significant effect, which can be additionally strengthened by optimizing the value of the parameter $S_{CW}$.

The results presented in Fig. 8, Fig. 9, confirm the conclusion just made. Analyzing the results presented in Fig. 8, one can see a linear relationship similar to $p_{ST}=f\left(\eta_{BS},S_{CW}\right)$ (see Fig. 6) between the synchronous growth of the average connection speed E(V) and the coverage density $\eta_{BS}$. At the same time, the density of the obstacle appearance intensity $\eta_{O}$ causes a very noticeable effect on the value of the characteristic E(V): an increase in the value of $\eta_{O}$ from 0.1 to 0.7 caused a drop in the value of E(V) by 5 times, regardless of the value of $\eta_{BS}$ (this can be explained by the fact that, under the condition that obstacles are distributed over the area $R^{2}$ evenly, therefore, affect both the information environment of network segment $NS_{L}$ and the information environment of network segment $NS_{\bar{L}}$). Finally, from Fig. 9, it can be seen that the reduction of the total probability of loss of requests Q depends not so much on the coverage density $\eta_{BS}$, but on the value of the coefficient 0<η≤1, which regulates the ratio between $S_{L\bar{L}}$ and $S_{\bar{L}}$ requests (the larger η, the greater the share of $S_{\bar{L}}$ requests), the support of which does not require compliance with the requirements QoS, however, is possible only in the network segment $NS_{\bar{L}}$.

In general, the obtained results showed the high efficiency of the approach of forwarding unregulated QoS requests to the unlicensed high-frequency range. The effect of such actions increases with the growth of the coverage density of the target area. It should also be noted the sensitivity of all qualitative performance metrics of the investigated 5G network to such parameters as the density of the obstacle appearance intensity and the value of the Contention Window as an important parameter of the Channel Observation-Based mechanism. There is a prospect of setting tasks for optimizing the target quality characteristics of the investigated 5G network relative to the values of these parameters.

## 5 Conclusions

Economic expediency encourages mobile operators to deploy 5G networks in places with a high concentration of fast-demanding subscribers (in frequented public places, areas with high-rise buildings, etc.). In such conditions, sharp fluctuations in the volume of traffic with regulated requirements for the quality of service are inevitable. Note that 5G operates in the millimeter range. Accordingly, the quality of traffic service is affected both by the number of subscribers simultaneously initiating requests from one sector of coverage, and by the appearance of obstacles opaque to radio radiation in the space between the subscriber device and the base station. Effective smoothing of 5G traffic fluctuations, taking into account these disturbing factors, is an urgent task.

To solve the problem, the article proposes to use such technologies of the 5G platform as Network Slicing, New Radio Unlicensed, and Carrier Aggregation to be used in the process of servicing requests for both licensed (28 GHz) and unlicensed (38 GHz) spectrum of the millimeter range. Protocols operating in the unlicensed frequency range use the random access procedure, accordingly, they cannot guarantee the communication speed regulated by the quality of service requirements. This limitation can be mitigated by effectively controlling the density of base stations, calculated to support a corresponding density of subscriber devices capable of functioning in the entire millimeter range of 5G.

The article investigates the Markov process of simultaneous maintenance of sessions by 5G base stations in network segments deployed in licensed and unlicensed frequency ranges. In terms of queuing theory, a model has been developed that allows to calculation speeds for sessions in the mentioned network segments for the target 5G network. These parameters are used as a basis for determining the probabilities of session loss in the mentioned network segments and for estimating the necessary base station placement density to support the traffic of a corresponding density of dual frequency range subscriber devices for the target coverage area of the 5G network. The model takes into account both the possibility of redirecting sessions to the network segment deployed in the unlicensed frequency range, as well as the impact on the quality metrics of the sessions of the disturbing factors mentioned above.

The results of the experiments showed that the probability of losing sessions with regulated requirements for the quality of service in both network segments, in addition to the density of placement of base stations and subscriber devices, is significantly affected by the minimum data transfer rate, the intensity of obstacles, and the value of the Contention Window. In general, the obtained mathematical apparatus allows us to adequately describe the functioning of the dual frequency range 5G network in the metric of the mentioned parameters, which is especially relevant at the stage of designing the latter.

Experiments have shown that it is in conditions of high intensity of the appearance of obstacles that the use of an unlicensed frequency range gives a significant effect, which can be enhanced by optimizing the value of the Contention Window in the parametric space of the created model. ***Further research*** is planned to be directed to the formulation of such an optimization problem and the investigation of its properties.

## CRediT authorship contribution statement

**Viacheslav Kovtun:** Writing – review & editing, Writing – original draft, Visualization, Supervision, Software, Project administration, Methodology, Investigation, Funding acquisition, Formal analysis, Conceptualization. **Krzysztof Grochla:** Writing – review & editing, Validation, Resources, Data curation. **Elena Zaitseva:** Writing – review & editing, Validation, Resources, Data curation. **Vitaly Levashenko:** Writing – review & editing, Validation, Resources, Data curation.

## Data availability statement

All relevant data are within the manuscript.

## Funding

This research is part of the project No. 2022/45/P/ST7/03450 co-funded by the National Science Centre and the European Union Framework Programme for Research and Innovation Horizon 2020 under the Marie Skłodowska-Curie grant agreement No. 945339. For the purpose of Open Access, the author has applied a CC-BY public copyright licence to any Author Accepted Manuscript (AAM) version arising from this submission.

## Declaration of competing interest

The authors whose names are listed immediately below certify that they have NO affiliations with or involvement in any organization or entity with any financial interest (such as honoraria; educational grants; participation in speakers’ bureaus; membership, employment, consultancies, stock ownership, or other equity interest; and expert testimony or patent-licensing arrangements), or non-financial interest (such as personal or professional relationships, affiliations, knowledge or beliefs) in the subject matter or materials discussed in this manuscript.
